# Memory-Efficient Deep Learning on a SpiNNaker 2 Prototype

**DOI:** 10.3389/fnins.2018.00840

**Published:** 2018-11-16

**Authors:** Chen Liu, Guillaume Bellec, Bernhard Vogginger, David Kappel, Johannes Partzsch, Felix Neumärker, Sebastian Höppner, Wolfgang Maass, Steve B. Furber, Robert Legenstein, Christian G. Mayr

**Affiliations:** ^1^Chair of Highly-Parallel VLSI-Systems and Neuromorphic Circuits, Department of Electrical Engineering and Information Technology, Institute of Circuits and Systems, Technische Universität Dresden, Dresden, Germany; ^2^Institute for Theoretical Computer Science, Graz University of Technology, Graz, Austria; ^3^Bernstein Center for Computational Neuroscience, III Physikalisches Institut – Biophysik, Georg-August Universität, Göttingen, Germany; ^4^Advanced Processor Technologies Group, School of Computer Science, University of Manchester, Manchester, United Kingdom

**Keywords:** deep rewiring, pruning, sparsity, SpiNNaker, memory footprint, parallelism, energy efficient hardware

## Abstract

The memory requirement of deep learning algorithms is considered incompatible with the memory restriction of energy-efficient hardware. A low memory footprint can be achieved by pruning obsolete connections or reducing the precision of connection strengths after the network has been trained. Yet, these techniques are not applicable to the case when neural networks have to be trained directly on hardware due to the hard memory constraints. Deep Rewiring (DEEP R) is a training algorithm which continuously rewires the network while preserving very sparse connectivity all along the training procedure. We apply DEEP R to a deep neural network implementation on a prototype chip of the 2nd generation SpiNNaker system. The local memory of a single core on this chip is limited to 64 KB and a deep network architecture is trained entirely within this constraint without the use of external memory. Throughout training, the proportion of active connections is limited to 1.3%. On the handwritten digits dataset MNIST, this extremely sparse network achieves 96.6% classification accuracy at convergence. Utilizing the multi-processor feature of the SpiNNaker system, we found very good scaling in terms of computation time, per-core memory consumption, and energy constraints. When compared to a X86 CPU implementation, neural network training on the SpiNNaker 2 prototype improves power and energy consumption by two orders of magnitude.

## 1. Introduction

The number of connections is the main limiting factor in the up-scaling of neural network implementations. It dominates the required chip area (Benjamin et al., 2014; Schmitt et al., 2017) and power consumption (Schemmel et al., 2008; Akopyan et al., 2015) in special purpose hardware and the required memory and computation time (Brette et al., 2007) in software simulations. When neural networks are used in embedded systems, e.g., for medical applications, robotics, or tactile devices, their physical realization needs to be extremely energy efficient to meet requirements on durability, size, and heat emission (König et al., 2002; Sze et al., 2017). These limitations have so far prevented the use of resource-intensive deep learning algorithms (LeCun et al., 2015) directly in embedded systems.

Here, we show an implementation of a deep neural network with learning capabilities on a neuromorphic hardware chip. Both the training phase and the inference phase are implemented on a 2nd generation SpiNNaker prototype. All the memory that is required for neuron and synapse parameters is kept in the fast, local 64 KB SRAM of the chip. To operate the network at such a low memory footprint, we use the recently introduced Deep Rewiring (DEEP R) model for training deep networks with sparse weight matrices (Bellec et al., 2018a). DEEP R searches for an optimal connectivity structure by dynamically cutting off and rebuilding network connections online. Therefore, despite the severe memory limitations on this hardware, optimization of neural networks with DEEP R can be implemented directly on-chip using sparse weight matrices. The presented implementation thus runs at a constant memory budget that is orders of magnitude smaller than that of a fully connected network. This innovation drastically cuts memory and computation time requirements at only a small performance loss. DEEP R has previously been applied to feedforward and recurrent networks with sparse weights as well as convolutional models with sparse feature maps. Here for the first hardware implementation of DEEP R, it is used to train a feedforward model with sparse weights under the strong memory limitation of a tiny four-core prototype.

Our implementation makes efficient use of the hardware accelerators that are available on the SpiNNaker prototype chip. The chip is equipped with high-throughput accelerators for fixed-point exponentiation and random number generation. Both operations are commonly needed in the neural network domain and are also used in the DEEP R algorithm. We show that using the hardware accelerators results in a speedup of around 1.62 × in our setup without any further software optimizations. Together with parallelization from one core to four cores, a total speedup of 7.7 × is achieved.

We evaluated our implementation of DEEP R on the SpiNNaker prototype chip using a benchmark network architecture for learning the MNIST benchmark dataset. The complete network can be simulated on a single core of the chip using < 40 KB of the SRAM (see section 3.1) and also on multiple cores for time speedup and higher energy efficiency purpose. In section 3.2 we study the computation time speedup that can be gained by using hardware accelerators and multiple cores. Finally, in section 3.3 we study the power and energy requirements of our system. In summary, our results demonstrate a full on-chip deep learning system, that achieves 96.6% accuracy on the MNIST benchmark dataset, using < 4% memory compared to a fully connected network, and only 2% of the power budget compared to running the same algorithm on an X86 processor.

## 2. Materials and methods

### 2.1. Hardware

SpiNNaker (Furber et al., 2014) is a digital neuromorphic hardware system based on low-power ARM processors built for the real-time simulation of spiking neural networks (SNNs). On the basis of the first-generation SpiNNaker architecture and our previous work in power efficient multi-processor systems on chip (Haas et al., 2016, 2017), the second generation SpiNNaker system (SpiNNaker2) is currently being developed in the Human Brain Project (Amunts et al., 2016). By employing a state-of-the-art CMOS technology and advanced features such as per-core power management, more processors can be integrated per chip at significantly increased energy-efficiency.

**Figure 1:**
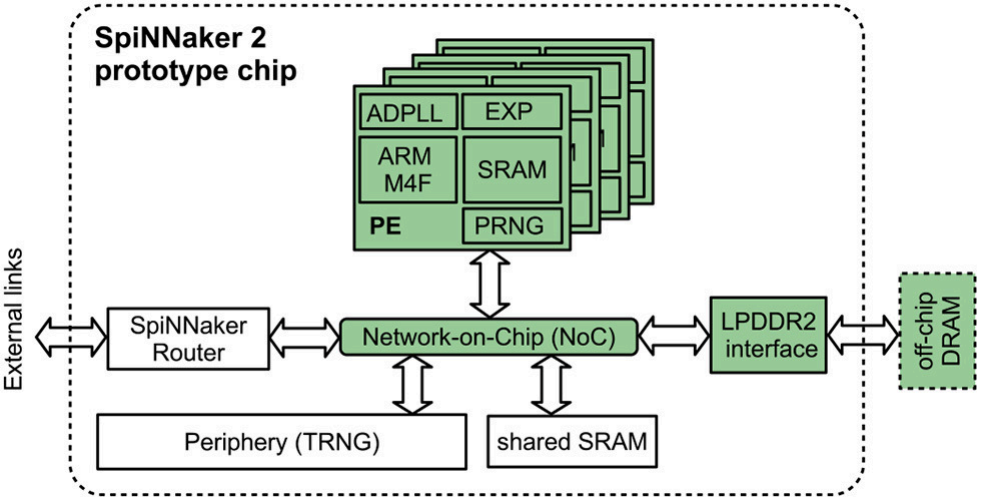
System overview of the SpiNNaker 2 prototype system. Components used in this article are highlighted in green.

In this article, we use the first SpiNNaker2 prototype chip which has been implemented in GLOBALFOUNDRIES 28 nm SLP CMOS technology (Höppner et al., 2017). Its system overview is shown in Figure 1: It contains four processing elements (PEs) with ARM M4F processors and 128 KB local SRAM (64 KB instruction, 64 KB data), an LPDDR2 interface to 128 MB off-chip DRAM, the SpiNNaker router for on- and off-chip handling of spike packets, and a network-on-chip (NoC) for the communication between all chip components. The system operates in a globally asynchronous locally synchronous (GALS) scheme, where each component has its own clock generator based on an all-digital phased-locked loop (ADPLL) (Höppner et al., 2013). Additionally, each PE contains high-throughput accelerators for the fixed-point exponential function (Partzsch et al., 2017) and pseudo random numbers (PRNG). A true random number generator (TRNG) in the periphery of the chip provides true randomness from silicon noise at a lower bandwidth (Neumärker et al., 2016) and a shared SRAM is also available to the PEs via the NoC. The above described features are not all used in this implementation. Only the components highlighted in Figure 1 are essentially involved in our implementation.

**Figure 2:**
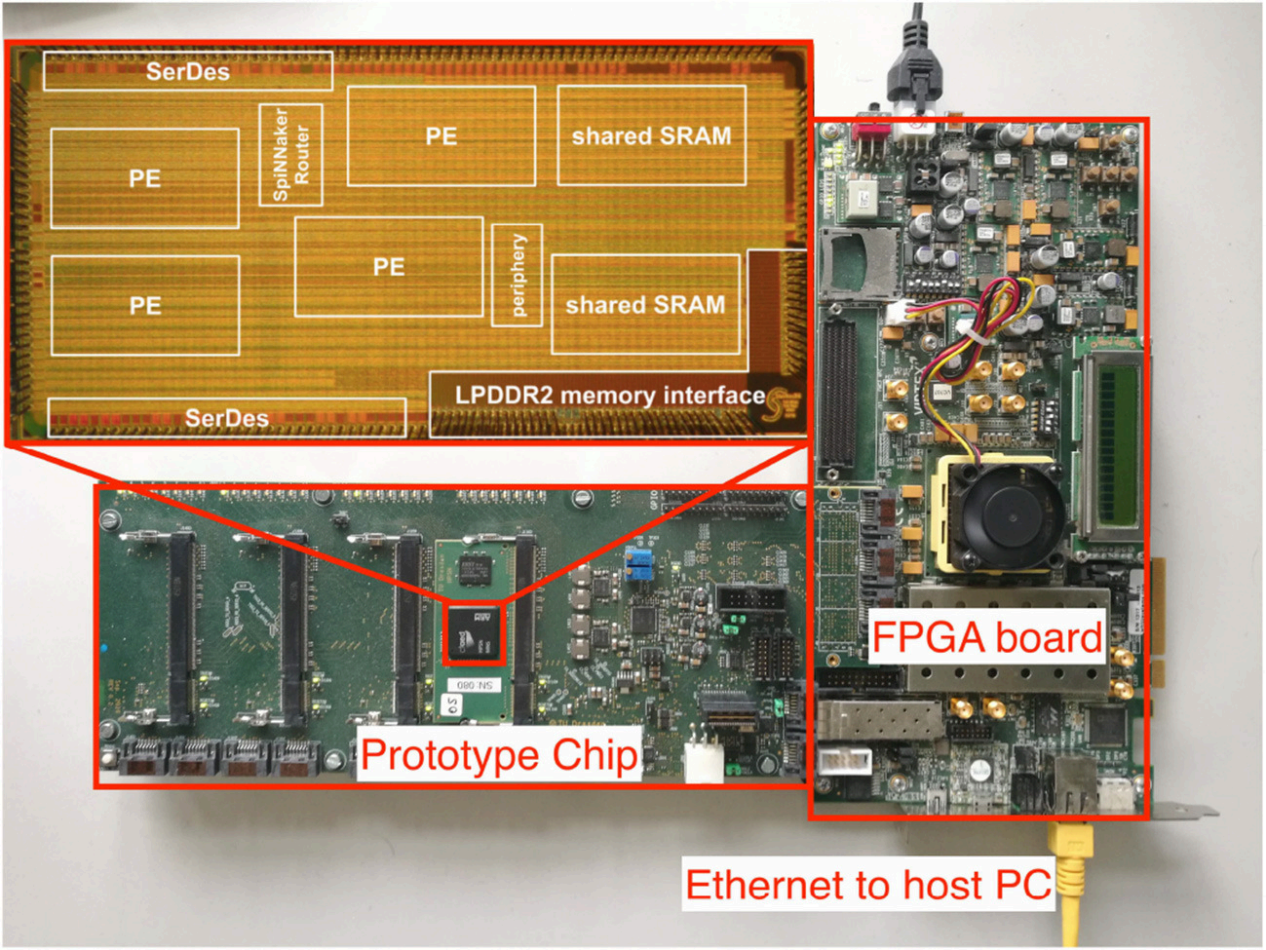
Photo of the entire setup and the prototype chip (Höppner et al., 2017). The setup consists of one SpiNNaker prototype chip hosted on a PCB board and an FPGA evaluation board. The chip photo is enlarged and placed at the top left corner.

A photo of our setup is shown in Figure 2. For lab evaluations, a power supply PCB is used which hosts up to four chip modules. This is connected to an FPGA evaluation board via serial links (SerDes) or JTAG which then connects to the host PC via standard Ethernet for debugging and result observing purpose.

### 2.2. MNIST learning task

**Figure 3:**
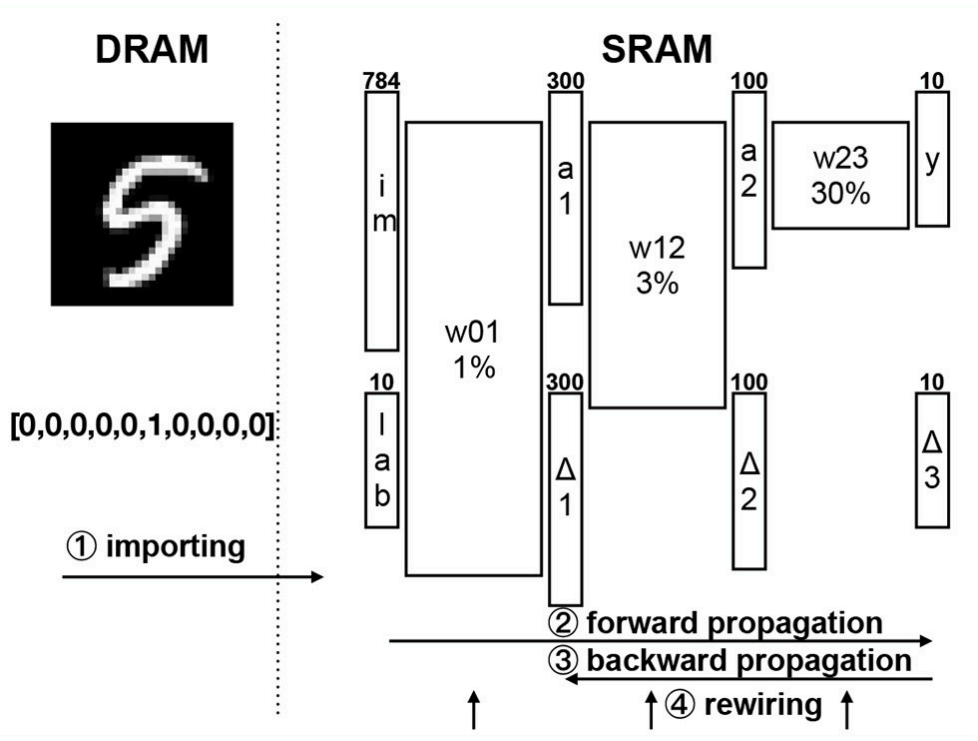
Details to the implementation of the neural network architecture and execution procedures. The network input and target data is held in the slow DRAM **(left)**. One example input image and binary coded target vector are shown. The network parameters are held in the fast SRAM **(right)**. Training images are presented to the input layer (im) with 784 channels (28 × 28 pixels). Network activations (*a*_*i*_) are propagated forward through the network and errors (Δ_*i*_) are propagated backward. The 10 neurons of the output layer (y) correspond to 10 classes of digits. The weight matrices stores the connection weights and related sparsity are marked with W plus percentage. The target (lab) is encoded with one-hot code. The circled numbers with arrows at the bottom present the order of execution procedures in one training iteration and their scopes.

We apply the DEEP R algorithm (see section 2.3) to train a network architecture that was previously chosen in Han et al. (2015) to benchmark memory efficient networks. As shown in Figure 3, this architecture has a 784-neuron input layer, two hidden layers with 300 and 100 neurons respectively, and a 10-neuron output layer. All hidden units use a rectified linear activation function. All layers are sparsely connected and connections are selected online using DEEP R. The connectivity of weight matrices (i.e., the percentage of non-zero matrix entries) is constrained to a fixed value of 1, 3, and 30% for matrices to hidden layers 1, 2, and to the output layer respectively. These choices follow the rule of thumb from Bellec et al. (2018a): the smaller the weight matrix, the more densely it is connected. This results in a fixed overall connectivity of 1.3%. Thus the memory footprint is significantly reduced compared to a fully connected architecture and weight matrices, activation vectors, errors, and gradients can all be accommodated in the 64 KB local SRAM, which means that the on-chip training is possible within a single core.

To benchmark the performance characteristics of the implementation on the prototype chip, we used the MNIST dataset (LeCun et al., 1998). It contains 60 k 256-level digital gray scale images, where each image has the size of 28 × 28 pixels. We reserved 50 k images as the training set and 10 k as the test set.

### 2.3. The DEEP R algorithm

In DEEP R, every potential connection of the neural network can at any time during training either be *active* or *dormant*. An active connection is actually realized, its parameters are stored in memory, and information is transmitted over this connection. A dormant connection is not realized at that moment, no parameters are stored in memory, and no information is transmitted.

The DEEP R algorithm alternates stochastic gradient descent (SGD) steps and rewiring steps. SGD updates are applied to weights of all active connections using error backpropagation, while the weight of dormant connections are by definition 0 and do not need to be stored or updated. In addition *L*_1_-regularization and gradient noise is applied to active connections in each SGD step. For each active weight that crosses zero (changes sign) after the SGD update, DEEP R sets this connection dormant and activates another connection that was dormant. New connections are randomly drawn from all possible neuron pairs within the layer with equal probability. The new connection is initialized with a weight of 0 and then evolves according to SGD updates.

It was proven in Bellec et al. (2018a), that this algorithm samples weights and network architectures (connectivities) from a well-defined posterior distribution that respects the global objective function underlying the SGD updates and the sparsity constraints. In other words, the algorithm is theoretically guaranteed to converge to a network configuration where the active connections and their weights are close to a minimum of the classification error and at the same time strictly obey the prior sparsity. To illustrate the sampling process of DEEP R, one can consider a newly replenished connection: if its gradients at the next time step predicts that this connection reduces the classification error, the SGD update is likely to set the weight apart from zero and the connection is kept; if this connection has no effect or is detrimental to the network function, the gradient—pressured by the *L*_1_-regularization—crosses zero in the very next time step, and disappears. As a result, only functional connections have a high chance to remain in the network.

DEEP R is implemented on the SpiNNaker 2 prototype chip in an online fashion, i.e., one input image is processed after another. A summary of the algorithm is given in Algorithm 1. The entire MNIST dataset cannot be stored in the SRAM. Instead, the images are placed in the external DRAM and only the image that is currently processed with its associated label are loaded into the SRAM via the LPDDR2 interface in the form of NoC packets. After each image importation (line 2 of Algorithm 1) the DRAM interface is turned-off to avoid unnecessary energy consumption.

**Algorithm 1:**
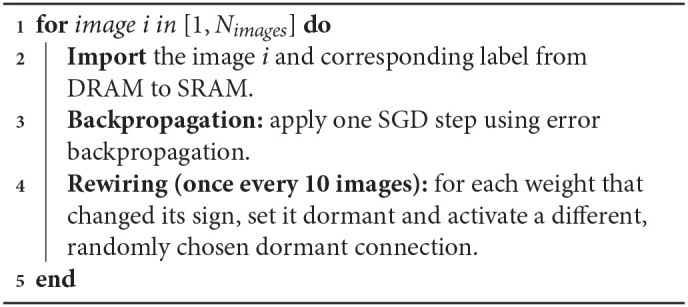
Pseudocode of the online version of the DEEP R algorithm implemented on-chip

Each backpropagation step at line 3 is split into a forward and a backward pass. In the forward pass—also called inference—the activation generated by an image is propagated from the first layer to the last through the network to predict the image label. In the backward pass, the predicted label is compared to the target label and the error is backpropagated from the last layer to the first. The learning rate η for SGD is initialized to 0.05 and decays by a factor of 0.5 every 2 training epochs. The *L*_1_-regularization parameter of DEEP R is set to 10^−5^. The temperature *T* for the gradient noise of the DEEP R algorithm is also annealed alongside the learning rate, i.e., $T=\eta \frac{\sigma^{2}}{2}$ where σ = 0.0003. For details on the initialization of sparse weight matrices see Bellec et al. (2018a).

The DEEP R algorithm ensures that the memory consumed by the network connectivity is constant and the memory requirement is a parameter of the algorithm that can be chosen to fit hardware limitations. We constrained the number of connections individually for each weight matrix, i.e., the number of connections between adjacent layers, such that each weight matrix consumes a fixed amount of memory on one particular core.

### 2.4. Sparse matrix formats and implementation details of the rewiring algorithm

To perform the rewiring step at line 4 of Algorithm 1, we delete all the weights that changed signs during the SGD step, and reconnect the corresponding number of connections elsewhere. The weights in each layer constitute a sparse weight matrix, and there exist several possibilities how such sparse matrices can be stored efficiently in memory. The choice of the matrix format was indeed important to optimize both memory consumption and speed. To optimize speed one has to take into account that within the forward and the backward passes of backpropagation it is required to multiply the sparse weight matrices from two different directions. In the rewiring step it is required to delete and insert elements efficiently.

**Figure 4:**
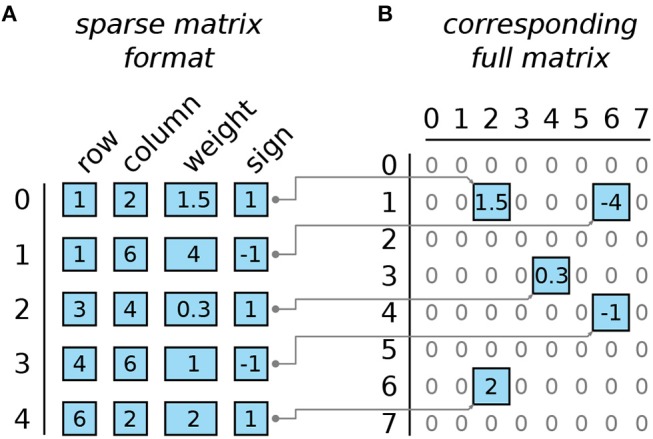
Illustration of the sparse matrix format. **(A)** Example matrix of size 8 × 8 with 5 non-zero entries encoded in the sparse matrix format. Each row encodes one entry. Entries are sorted by the row/column pair (row first, column second). **(B)** The full matrix that corresponds to the example sparse matrix in **(A)**.

The popular matrix formats, compressed sparse row (CSR) and compressed sparse column (CSC) first described in Tinney and Walker (1967), are inappropriate for backpropagation. They are optimized for either left or right side matrix-vector multiplication while multiplication from the other side is slow. We chose instead a variant of the compressed coordinate (COO) format, where each entry is stored as a tuple (row, column, weight amplitude, and weight sign) and the entries are stored as arrays sorted by row/column pairs (see Figure 4 for an illustration). Rows and columns are stored in 16-bit integer (int16) format, weight amplitudes are stored in 32-bit floating point (float32) format. To optimize the memory consumption we used arrays ordered by row and column coordinates which avoids storing one pointer per matrix element in comparison to linked lists. This saves 4 bytes per matrix element with most compilers. The drawback is that the rewiring step involves more computations but this was compensated by applying rewiring only every 10 iterations. The reasons for this choice are detailed in section 3.2.

The rewiring step is split into a deletion and an insertion step. The deletion step erases the entries of all weights that changed the sign during the last gradient descent step. The insertion step replaces the deleted coefficients by other randomly selected matrix entries. The deletion is performed by passing once through the four arrays shown in Figure 4. Each matrix coefficient that has a negative weight amplitude is deleted. Due to the data structure, this deletion is implemented by swapping entries in all four arrays until the deleted elements are pushed at the end of the arrays. Then the insertion step begins. Firstly, all deleted elements are replaced by new matrix coefficients with: random coordinates, zero weight amplitude, and a random binary sign. Secondly one needs to sort the entire matrix representation by row and column indices. One solution is to apply quicksort to sort the array as a whole. This method is rather fast but may overload the SRAM because quicksort requires the storage of temporary variables and the amount of such variables is hard to estimate. In practice, only the few dozens of newly inserted zero-elements which are now grouped at the end of the array are sorted with quicksort (we bounded the number of zero-elements that can be inserted in each step to avoid memory overload). At that stage the arrays are split into two sorted sections: the preserved matrix elements at the beginning, and the newly inserted elements at the end. Taking advantage that the two sections are already sorted correctly, the arrays are merged with an in-place variant of merge-sort.

### 2.5. Distributed implementation

The neural network we implemented in this article is relatively simple and can be fully stored in a 64 KB SRAM of one ARM core. But with the escalating complexity of the modern machine learning architectures, a network involving numerous parameters may not be accommodated in one machine. Also, a larger network results in more computation time and a larger memory footprint. It is thus necessary to discuss how to use multiple computation nodes to train a deep neural network. SpiNNaker 2 is a multi-core system that naturally features multi-core distributed implementations such that we employed the 4-core prototype chip to implement the above network.

#### 2.5.1. Distribution methodology

Neural network training algorithms are memory-intensive because they involve massive data sets and complex network architectures with large parameter sets. In terms of these two factors, there are in principle two strategies for parallel training (Shrivastava et al., 2017).

##### 2.5.1.1. Data parallelism

Data too big to fit into the memory of a single node can be partitioned across several nodes. The learning model is duplicated into each node and trained with different input data simultaneously. This approach maintains the model structure in each node and facilitates rapid deployment across multiple nodes.

##### 2.5.1.2. Model parallelism

If network (or model) parameters cannot be accommodated in a single node, they can be split into several sub-modules and stored in different nodes. In this case the same data is fed into different nodes, but on each node only a subset of the model is trained. In other words, the computation of the entire model is separately allocated on the distributed nodes.

The network structures considered in this article are feedforward neural networks. Although the connections between adjacent layers are sparse, the memory is dominated by the weight matrices and not by the neuron activities (see section 3.1). With data parallelism, all weight matrices are duplicated in each node. On the other hand, model parallelism is well-suited to distribute large models where model parameters dominate the memory consumption. Therefore, we did not adopt data parallelism but model parallelism as the implemented distribution methodology.

#### 2.5.2. Partition scheme

The performance improvement of model parallelism depends directly on how the model is split. A good model partition scheme should balance the computational load of each node while minimizing the data dependency between nodes.

**Figure 5:**
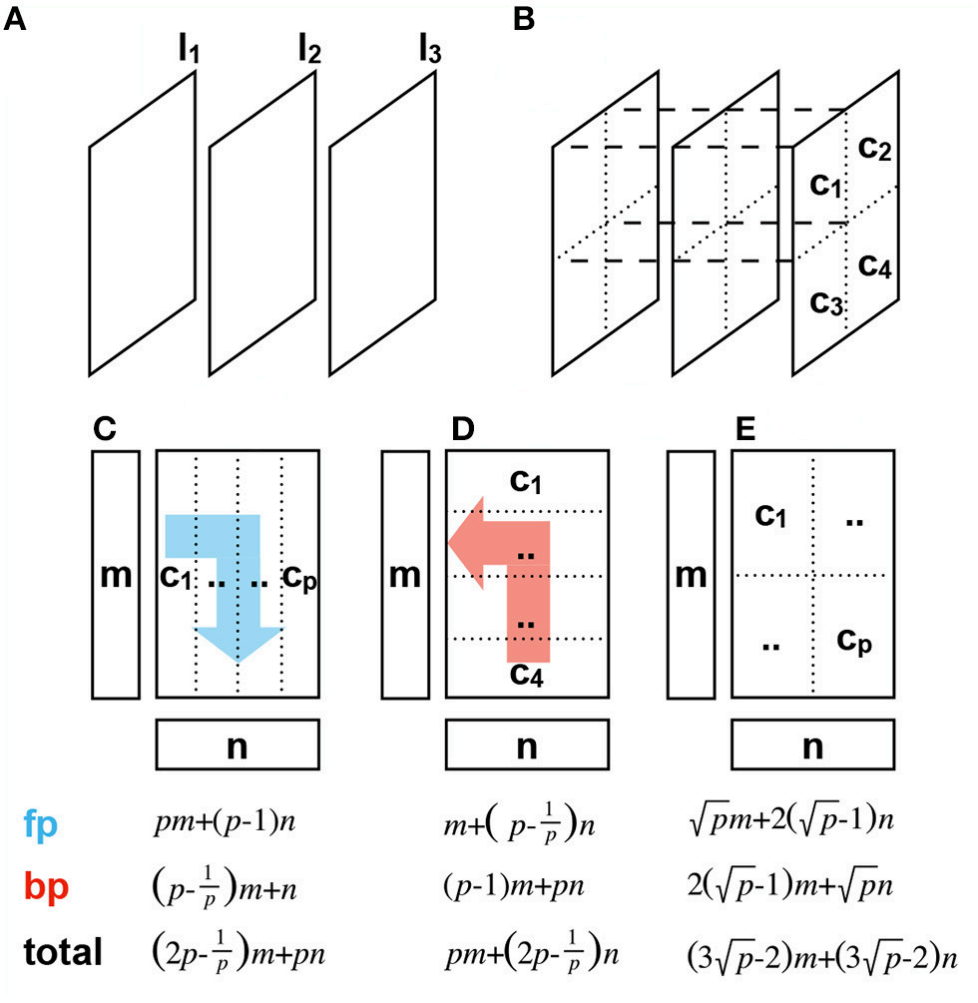
Different partition schemes and their communication overhead analysis. The forward pass (fp) and backward pass (bp) directions are marked in blue and red, respectively. Given that the matrix has a size of *m*×*n*. **(A)** Layer-wise partition stores one layer in one node, such as *l*_1_, *l*_2_, *l*_3_. **(B)** Channel-wise partition stores one channel in one node, such as *c*_1_, *c*_2_, *c*_3_, *c*_4_. **(C)** 1D vertical channel-wise partition. **(D)** 1D horizontal channel-wise partition. **(E)** 2D channel-wise partition.

Figures 5A,B illustrate 2 different partition schemes. If the model is layer-wise partitioned, each layer of a deep neural network is deployed in one node and all nodes form a pipeline structure. Only the activities in the forward pass and errors in the backward pass are passed between nodes. However, the size of different layers can vary dramatically so that the required amount of computation on different nodes also differs significantly. This unbalanced utilization of resources among nodes can evidently hamper the execution efficiency of the pipeline.

Another partition scheme is channel-wise partitioning, under which each node stores partial parameters of one layer. These partial parameters throughout all layers are combined to form a channel. Whether in forward pass or backward pass, to compute the output of one layer multiple nodes need to communicate with each other and broadcast their intermediate results because each node has only partial parameters and cannot complete the computation alone. The channel-wise partition makes distributed computing independent of the network structure so it has a better adaptability to different network depths or layer sizes, and the computational load is evenly distributed on each node to maximize the use of resources. We adopted the channel-wise partition to implement the distributed training so the weight matrices, various activations, errors, and gradients are deployed on different nodes and are updated locally.

To be more specific, the channel-wise partition has also several variants. If we focus on the weight matrix, the most computation-intensive operation in backpropagation is essentially the vector-matrix multiplication of the form *y* ← *Ax*. To perform the vector-matrix multiplication in parallel, we decompose the input vector and the matrix into multiple sub-vectors and sub-matrices and map them into different computation nodes for simultaneous calculation. Finally, the results on each node are restored to the original result. The matrix can be partitioned into multiple column vectors, row vectors, or sub-matrices (Uçar and Aykanat, 2005). Figures 5C–E depicts the vertically and horizontally one-dimensional (1D) channel-wise partition and two-dimensional (2D) channel-wise partition. We assume that the input and output vectors have the length of *m* and *n*, respectively. *p*, which is a square number, computation nodes are available.

We analyze the communication pattern in the forward pass under 1D vertical partition as an example. Firstly, the input vector is duplicated onto *p* nodes, the transmission amount is *pm*. After the completion of vector-matrix multiplication on each node we obtain the output vector component *n*_*i*_, *i* ∈ [1, *p*] dispersedly. Then the vector components are synchronized among the nodes to prepare for the forward propagation for the next layer. For instance, the core *c*_1_ sends the vector component *n*_1_ with the length of $\frac{n}{p}$ to core *c*_*j*_, *j*∈[2, *p*], which brings a communication overhead of $\left(p-1\right)\frac{n}{p}$. Therefore, *p* nodes delivered (*p*−1)*n* data. Thus, in the 1D vertical partition, a forward propagation in one layer transfers *pm*+(*p*−1)*n* data. The multifarious communication overheads in forward and backward pass with 1D and 2D partitions are listed at the bottom of Figure 5. For one layer we found that since the forward pass involves right dot multiplication while the backward pass involves left dot multiplication, 1D partitioning always rewards one and punishes the other. In contrast, 2D partitioning can primely balance the communication overhead in both directions and reduce the communication complexity from *O*(*p*) to $O\left(\sqrt{p}\right)$. This so-called checkerboarding 2D partition method is proved to be more efficient than 1D partition (Kumar et al., 1994).

#### 2.5.3. Distributed training on the prototype chip

**Figure 6:**
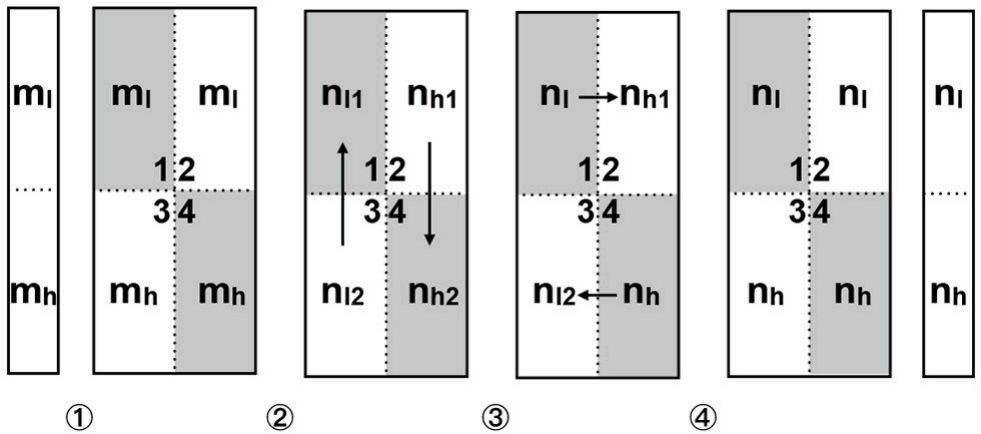
Data flow in 4 cores for parallel vector-matrix multiplication. The diagonal cores (core 1, 4) are highlighted in gray. ①Importing *m*_*l*_ into core 1,2 and *m*_*h*_ into core 3,4. ②Vector-matrix multiplication in each core. ③Non-diagonal cores vertically send local results *n*_*l*2_,*n*_*h*1_ to diagonal cores and merge the local results there to *n*_*l*_ and *n*_*h*_. ④Diagonal cores horizontally broadcast the output vector components to non-diagonal cores (core 1 to core 2, core 4 to core 3).

We adopted the checkerboarding partition scheme to partition the network model. *p* is instantiated to 4 since 4 cores are available. The first weight matrix with the size of 784 × 300 is split into four 392 × 150 sub-matrices. Analogously this procedure generates 4 sub-matrices of size 150 × 50 for the second matrix and 50 × 5 sub-matrices for the third. All the activations and error vectors are equally partitioned into a high-end fraction and low-end fraction. Figure 6 shows how a vector-matrix multiplication is performed in parallel on the prototype chip. In the first step, the high-end fraction *m*_*h*_ and low-end fraction *m*_*l*_ of the input vector are loaded into 2 cores respectively. In the second step, each vector fraction is multiplied by the local weight sub-matrix, obtaining 4 vector components *n*_*l*1_, *n*_*l*2_ and *n*_*h*1_, *n*_*h*2_. In the third step, the non-diagonal cores vertically send the local vector components to diagonal cores and merge them with their local vector components to get the output vector's high-end fraction *n*_*h*_ and low-end fraction *n*_*l*_, e.g., in core 1 we gathered *n*_*l*_ = *n*_*l*1_+*n*_*l*2_. In the fourth step, the diagonal cores horizontally synchronize the vector fractions to non-diagonal cores, so that the low-end fraction *n*_*l*_ locates in core 1, 2 and the high-end fraction *n*_*h*_ in core 3, 4. At this time the vector fractions in each core are ready for the vector-matrix multiplication of the next layer.

Unlike the vector-matrix multiplication, the rewiring step involves matrix element addressing, removing, appending and sorting in a sparse matrix space. The parallelization does not negatively affect the speed of these operations. Instead it improves element addressing and sorting because the splitting of one big weight matrix into 4 quarter matrices reduces the lengths of the arrays represented in Figure 4. Yet, as the constraints are defined on quarters of matrices, there is less freedom in the choice of the position of the non-zero coefficients. This could in principle affect accuracy but we did not experience any loss of accuracy in practice. The acceleration result will be exhibited in Figure 10.

## 3. Results

We implemented a benchmark neural network architecture, previously used in Han et al. (2015), on the next-generation SpiNNaker prototype chip shown in Figure 2. This neural network architecture was previously used to benchmark the accuracy of sparsely connected low-memory footprint networks (Collins and Kohli, 2014; Han et al., 2015). Note that in these works, the network had a low-memory footprint only after training, while the training procedure itself is memory intensive. Here, for the first time, we *train* this architecture on a hardware with very restrictive memory constraints.

To train the network on the prototype chip with only 64 KB of SRAM memory per core, we used the DEEP R algorithm (Bellec et al., 2018a), which maintains a small memory footprint throughout training by dynamically disconnecting and reconnecting network connections (see section 2 for details).

**Figure 7:**
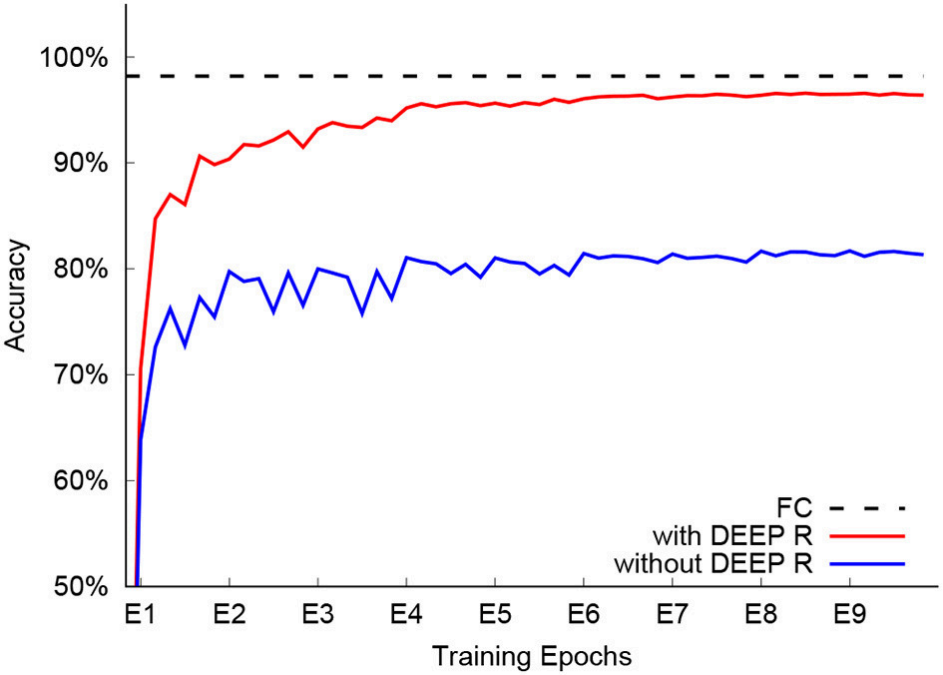
Classification accuracy of different setups. The accuracy of a fully connected network (FC, dashed line) is 98.2%, while sparsely connected networks (1.3% connection probability) achieve 96.6% with DEEP R (red) and 81.3% without online rewiring (blue).

Network training with the fixed connectivity of 1.3% converged in 9 epochs with a classification accuracy of 96.6% on the test set (see Figure 7). This is 1.6% below the baseline performance of 98.2% achieved by a fully connected network that cannot be trained on the chip. For comparison, we trained another sparsely connected network on-chip where we initialized the network connections from the same distribution that was found by DEEP R (see Bellec et al., 2018a) but did not rewire connections during training. The network performed significantly worse, reaching a classification accuracy of only 81.3%. This comparison shows that on-line rewiring is crucial for good network performance. For further comparison, since the 1.3% connectivity underutilizes the available memory in the 4 core setup (4PA case), we also tried fully utilizing the memory in the four processors. This way, we can go up to 9.3% connectivity and achieve 97.7% accuracy on the test set.

In the following, we analyze the memory profile, time consumption, power and energy consumption of the implementation based on 1 core and 4 cores of the prototype chip respectively.

### 3.1. Memory footprint

**Figure 8:**
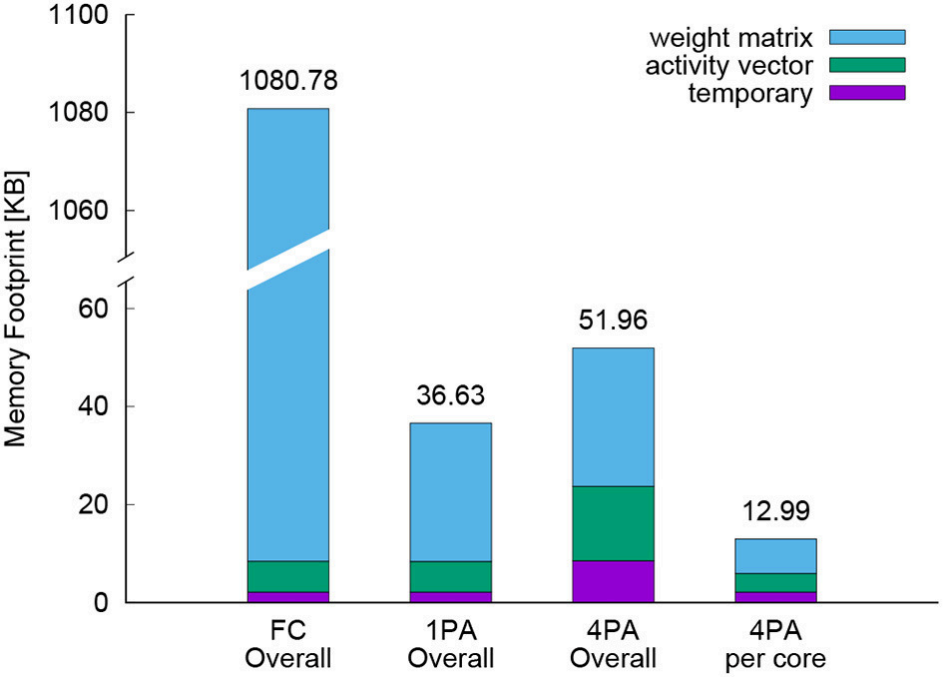
Memory profile of a fully connected network (FC Overall) and a sparse rewired network with one-core (1PA Overall) and four-core implementations (4PA Overall). Here number plus P indicates the utilized processor numbers and A stands for using hardware accelerators. For FC, the weight matrices, activity vectors, and temporary storage occupy 1072.34, 6.28, and 2.14 KB, respectively. In 1PA, there is no change on vectors and temporary storage but the weight matrix portion shrinks to 28.21 KB. In the four-core distributed implementation, the overall memory requirement of temporary storage, activity vector and weight matrix rise to 8.56, 15.16, and 28.24 KB. Dividing these values by four is the used resources in each core, which is indicated with 4PA per core. The total memory footprint in each case is shown above each bar.

Figure 8 shows the memory footprint for the fully connected network, the sparse network optimized with DEEP R, the same network with a training procedure parallelized over four cores and also visualizes the memory snapshot of one of these four cores. Memory consumption is divided into three components: the memory for the weight matrices, memory for activity vectors (including memory needed for backpropagated errors), and temporary storage. Here, temporary storage includes memory needed for dynamic function calls.

The DEEP R algorithm reduced the total memory footprint from 1080.78 KB down to 36.63 KB (with the small decrease in the performance described above). This enabled us to perform full training on a single core of the prototype chip with its limited SRAM of 64 KB. The weight matrices dominate memory consumption in all scenarios. Comparing the weight matrix portion between FC and 1PA, the sparse connectivity is 1.3% while the memory footprint percentage is 28.21/1072.34 = 2.6%, because storing the position of a sparse weight with COO format doubled the consumed memory.

The 2D channel-wise parallelization allocates the sub weight matrices into different cores, so there is no change on the overall volume of weight matrices, but it increased the activity vectors storage with 2.4 × and quadrupled the temporary storage. Two of this 2.4 are attributed to the duplicated storage of activity vectors by parallelization and the rest 0.4 come from some additional memory overhead of core-core communication. All these factors raised the overall memory consumption from 36.63 up to 51.96 KB. However, under 4-core implementation the available SRAM is also grown from 64 up to 256 KB, so the memory constraints are actually alleviated. For visualization, the memory footprint of one core under parallelization is depicted as the rightmost bar in Figure 8. Comparing the 2nd and 4th column, the SRAM utilization per core reduced from 57.2% (36.63/64) down to 20.3% (12.99/64).

### 3.2. Computation time

**Figure 9:**
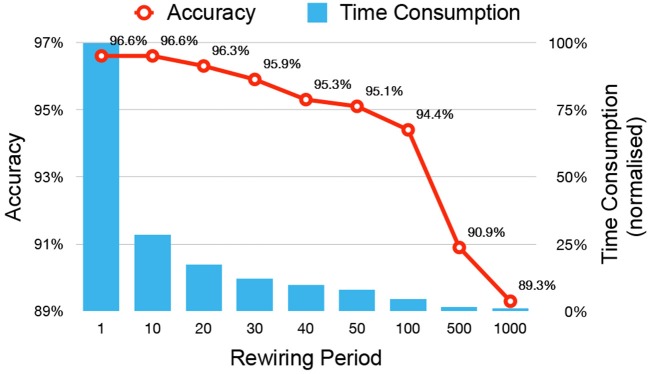
Accuracy and time consumption of applying rewiring with different periods. The iteration number between 2 rewiring operations is defined as the rewiring period. The time consumption is normalized by the time required with a rewiring period of 1 and depicted with percentage.

In a vanilla implementation of DEEP R with rewiring at each update step, the time needed for image data import, forward pass, backward pass, and rewiring accounted for 0.2, 2.5, 7.2, and 90.1% of the total computation time, respectively. Rewiring is the most time intensive step, because randomly replenishing new connections involve random number generations and sorting of matrix entries in the chosen memory-efficient sparse matrix format (see section 2.4). To improve runtime, we refined the algorithm such that it performs rewiring operations in only a fraction of the iterations. We refer to the number of iterations (i.e., parameter updates) between two consecutive rewiring operations as the rewiring period. Figure 9 shows the dependence of the accuracy and time consumption on the rewiring period. When the period is 10, no accuracy loss is observed, but the overall training speed is accelerated by a factor of almost 4. Further increasing the period value will decrease the network accuracy for the same number of training epochs. We chose a rewiring period of 10. At this rewiring period, the consumed time proportion of image data import, forward pass, backward pass, and rewiring steps are 1.0, 13.4, 38.0, and 47.6%, respectively, so that the time consumption of rewiring is comparable with that of the two passes of backpropagation.

On the SpiNNaker 2 system, hardware accelerators are available for random number generation and exponential function. Therefore, to perform any of these two operations, the ARM core can directly invoke the corresponding hardware module, consuming considerably fewer clock cycles, and less energy. In the discussed application, initialization of the weight matrix, generation of new connections, and noisy gradient updates involve random processes, and the softmax function in the last layer includes exponential operations. We therefore compare below the training time of the system with hardware acceleration and a pure ARM implementation that does not make use of these hardware accelerators.

The SpiNNaker 2 system is designed as a massively parallel processing network with many parallel cores that communicate via lightweight messages. Parallel training using multiple cores does not only allow for more complex networks, but is also expected to reduce training time. We tested parallelization of network training in the SpiNNaker 2 system, where we channel-wise partitioned the model into 4 channels and deployed them to 4 processors of the prototype chip.

**Figure 10:**
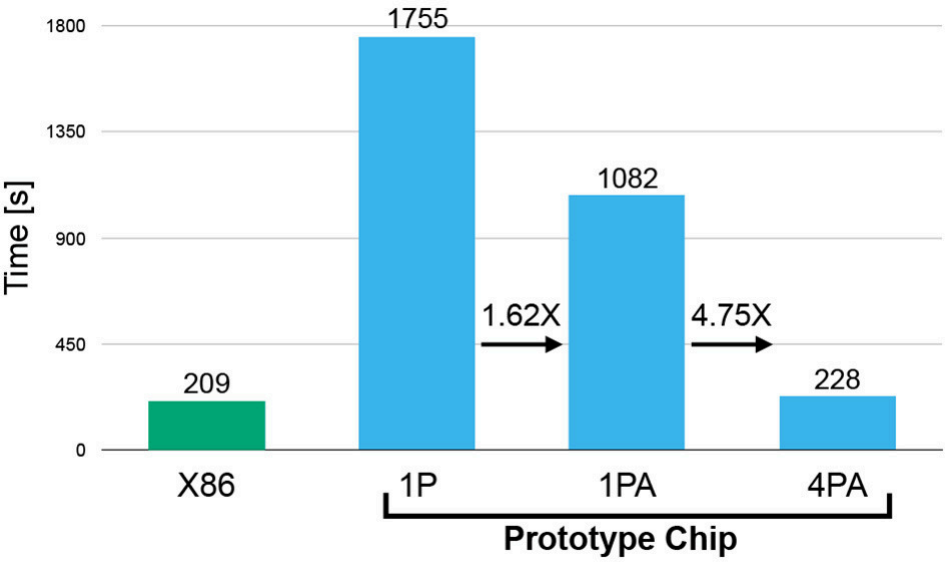
Time comparison on X86 platform and on SpiNNaker prototype chip with different setups. 1P indicates that 1 processor is utilized running pure ARM software, 1PA stands for 1 processor with hardware-based acceleration for random number generation and exponential computation, and 4PA stands for a 4-core parallelization with hardware acceleration.

Training time was evaluated in four setups (see Figure 10). First, we considered training (of a sparse network with DEEP R) on a X86 architecture (Intel Core i5 6500: 3.2–3.6 GHz; single core). This setup should serve as a baseline. Note that the clock frequency of this chip is more than 6 times higher than that of the SpiNNaker 2 prototype chip (500 MHz). Second, we considered an implementation on a single core of the SpiNNaker 2 prototype chip without hardware acceleration modules (1P). Figure 10 shows that the SpiNNaker implementation is about 8 times slower than the X86 implementation. However, when considering time consumption on a per-clock-cycle basis, we find that the SpiNNaker 2 prototype implementation is only slightly less efficient. Third, we considered a single-core implementation on the prototype chip with acceleration modules (1PA) for random number generation and exponential operation. The former is frequently carried out in the rewiring step and the latter is involved in the softmax function in the output layer. These special hardware features on the prototype provide an acceleration of 1.62 × , which decreased the training time from 1,755 down to 1,082 s. Finally, we considered a 4-core parallelized implementation on the SpiNNaker 2 prototype (4PA). In comparison with the single-core implementation (1PA), the training time drops from 1,082 to 228 s, which is only 21% of the original and is basically the same as the training time on the X86. It might seem surprising that the 4.75 × acceleration even outperforms the increment of processors. In fact, the parallelization strategy splits each weight matrix into 4 smaller matrices which are constrained to keep 4 times less non-zero coefficients during rewiring. This reduces the number of operations required by the sorting algorithms in the rewiring step. This result indicates that training of sparse neural networks with DEEP R can be highly parallelized on the SpiNNaker 2 system, with good speedup and per-core memory reduction. While we focus on the overall time course of the learning in this section, for comparison to other work (O'Connor et al., 2013; Stromatias et al., 2015) it is also interesting to give a perspective of a single MNIST image feedforward inference, which takes 0.27 ms in the 1 PA implementation and 0.1 ms in the 4 PA implementation.

### 3.3. Power and energy consumption

The profiled power consumption on the two platforms is listed in the first row in Table 1. We used the running average power limit (RAPL) energy sensor, which is a new feature provided by Intel since the Sandy Bridge micro-architecture (Hähnel et al., 2012), to measure the power consumption of the X86 CPU. The power of the prototype chip was determined by reading the supplied voltage and current from an ADC on the prototype PCB board. The power consumption of the X86 CPU in idle and running state was measured as 800 and 20,500, mW respectively. In the idle state, the CPU is powered on, maintaining the operating system, while in running state the training is performed.

**Table 1:** Power and energy consumption of neural network training with DEEP R on an X86 platform and the SpiNNaker2 prototype with one (1P, 1PA) and four (4PA) cores.

	**X86**	**Prototype 1P**	**Prototype 1PA**	**Prototype 4PA**
Power [mW]	20,500	88	88	227
Time [s]	209	1,805	1,082	228
Energy [J]	4,285	159	95	52

The prototype chip can be configured in a fine-grained form so that we could selectively activate different modules of the chip in different execution phases. The whole training execution could be divided into two phases: import (step 1 in Figure 3) and training (step 2, 3, 4 in Figure 3). In the import phase, the input data (images and labels) are loaded from the external DRAM via the LPDDR2 interface into the SRAM but the ARM core does not process them. In the training phase, the ARM core processes the data in SRAM and we close the LPDDR2 interface since no data flows from DRAM and we could save 46.4 mW. Note that the import phase accounted for only 1% of the total execution time, so that the average power consumption could be approximated as the training power. The power consumption during training was 87.9 mW in the 1P and 1PA (one core without/with accelerators) setups and 227.0 mW in 4PA (4 cores with accelerators) setup. This is a power reduction of 2 orders of magnitude compared to the X86 CPU implementation.

Considering the differences of computation time and power consumption on both platforms, Table 1 compares the energy required for the entire training process on a single core of the X86 platform and the prototype with either 1 or 4 cores. Profiting from the natural low-power characteristic of the ARM core and the minimized external DRAM accesses, the overall energy consumption in the SpiNNaker2 system with 1 core (1P) is only 159 J, which is 3.7% of the energy consumed on the X86 platform. Incorporating the integrated acceleration modules on the core (1PA) further reduces energy to 95 J, which is 2.2% of the X86 platform. With 4 cores (4PA), the energy consumption further drops to 52 J, only taking 1.2% of the consumed energy on the X86 platform. The energy benefit of parallelization is contributed to the over-expected time acceleration with a factor of 4.75, while the running power is only increased to 2.6 times of the one-core implementation. Please note that the power values for the SpiNNaker2 prototype include some baseline power (leakage, infrastructure) that is independent of the number of switched-on cores. Therefore, the increase in power from 1 to 4 cores is less than a factor of 4.

For comparison of the energy efficiency with other hardware systems in section 4, we estimate that it takes around 7,500 FLOPs (7 k for multiply-accumulation, 500 for ReLU) for each digit classification, which consumes 23 μ*J* on both 1PA and 4PA setups. Thus the effective energy efficiency of 0.33 GFLOPS/W can be derived as the reciprocal of the energy per operation.

## 4. Discussion

In the classical von Neumann architecture, computing time and energy consumption is dominated by memory access. Memory access is particularly costly in this computing paradigm, due to the von Neumann bottleneck. By using a massively parallel architecture, the brain avoids the von Neumann bottleneck. This is believed to be one important factor contributing to the extraordinary energy efficiency of the brain and mimicked in neuromorphic hardware. However, in massively parallel architectures, communication is still a bottleneck (now, not between the central processor and the memory, but between the parallel processors or neurons). This is the case for both, the brain, and artificial neuromorphic systems. The brain volume is dominated by white matter, that is, by long-range connections between neurons. In the SpiNNaker system, there is a communication bandwidth limit between the parallel cores. But an even more severe bottleneck in the neural network implementation considered here is the local memory needed to store synaptic parameters. In other words, biological and artificial information processing systems face the same problems in one or the other way. The DEEP R algorithm was inspired by the way how this problem is tackled in the brain, that is, by an ongoing rewiring of connections. This uses the available resources in a flexible way such that network structure can be adjusted to the task demands. We have shown in this article that this biological principle is also applicable to artificial neuromorphic systems.

### 4.1. Scaling analysis

We showed that the SpiNNaker 2 prototype chip can be used for handwritten digit classification as exemplified by the MNIST dataset. The network used is a fully connected feedforward network whereas a convolutional network is necessary to achieve state-of-the-art performance on interesting computer vision problems, and recurrent networks are necessary to tackle speech recognition. In Bellec et al. (2018a), DEEP R has already proven its practicability on the CIFAR-10 image dataset with convolutional networks and on the TIMIT speech recognition dataset with recurrent LSTM networks. For an acceptable drop of 2% of accuracy on CIFAR-10, the global number of non-zero parameters in the network were cut down to 5%. Similar performance was achieved with LSTM networks on TIMIT. In Bellec et al. (2018a), DEEP R was used to reduce further the memory footprint of the convolutional networks by constraining the number of non-zero coefficients inside the feature maps. Nevertheless, those sparse networks are still too large to be implemented on the current SpiNNaker 2 prototype which has only 4 cores and thus 4 × 64 KB of memory. Based on these small-scale prototypes, the hardware roadmap in the Human Brain Project foresees a large-scale SpiNNaker 2 chip containing ca. 160 cores around 2020. This chip will be used to build up the 10 Mio core final SpiNNaker 2 system, capable of a significantly higher level of parallelism and thus performance than the prototype used here. In the following scaling analysis, we analyze the memory required for the implementation of the CIFAR and TIMIT networks of Bellec et al. (2018a) with the described approach. This gives us a lower bound for the number of cores on which the network has to be distributed on the final SpiNNaker 2 system.

**Figure 11:**
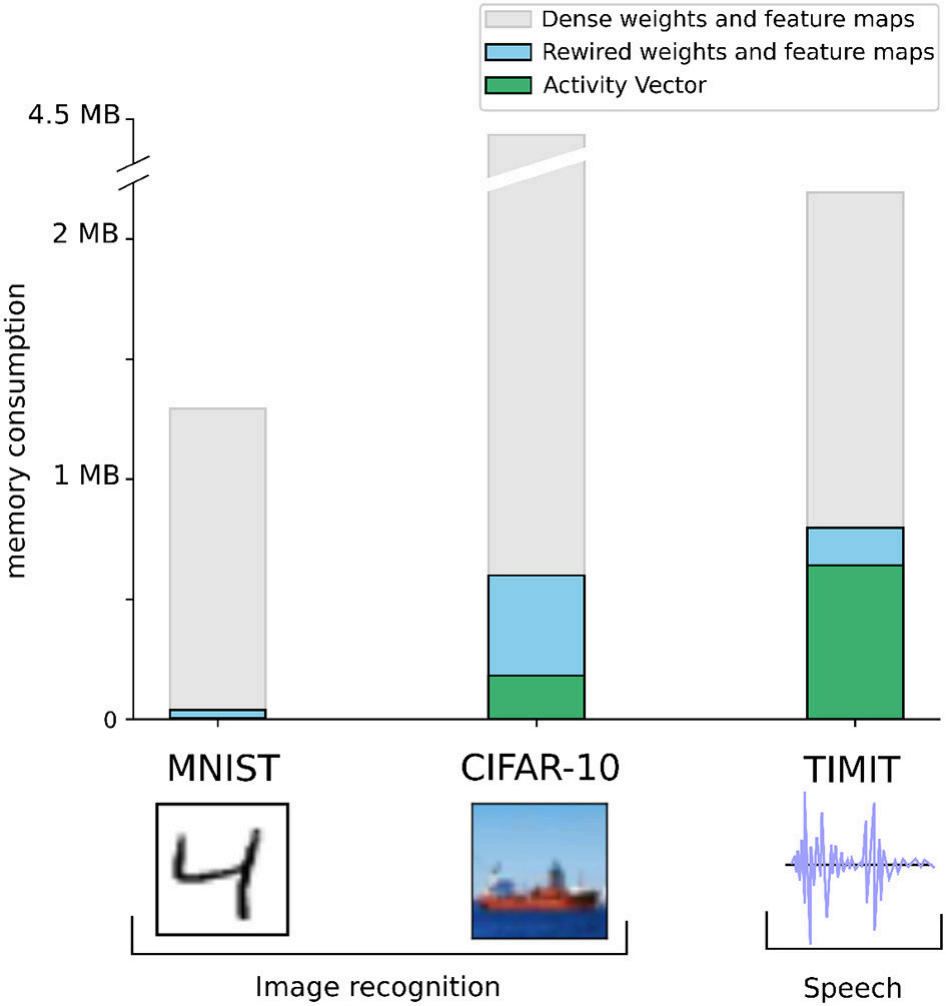
Scaling-up to real-world images and speech recognition. To study the importance of DEEP R on larger problems we evaluate the memory consumed by the storage of parameters in blue and the activity vectors required for backpropagation in green.

We first evaluate the memory requirements of the convolutional network used in Bellec et al. (2018a) on CIFAR-10. As demonstrated for MNIST in Figure 8 the memory is mainly occupied by the weight matrices and the intermediate activation of each layer which need to be stored for backpropagation. In this scaling analysis we consider that the storage of other temporary variables is negligible. For each connection of the fully connected layers, one has to store a row/column index, weight, and sign. This leads to 65-bits per parameter. In a convolutional layer, the convolutional kernel are indexed by 4 integers *i, j, k, l* where *i, j* are the row and column indices of the feature map which are lower than 5, and *k* and *l* are the input and output channels of the convolution which are bounded by 64. Storing each of the four integers on 8-bit, 1-bit for the sign and adding 32-bit to store of the real-valued weight, each parameter of a convolutional feature map is also stored on 65-bit. Thus the number of bits required to store the network parameters is 65 × *n*_*c*_×*p* where *n*_*c*_ is the number of parameters and *p* = 5% is the number of non zero parameters which were shown to be required to train a convolutional network on CIFAR-10 with DEEP R (Bellec et al., 2018a). The network was constituted of two convolutional layers with feature maps of shape 5 × 5 × 3 × 64 and 5 × 5 × 64 × 64 and two fully connected layers with matrices of shape 2, 304 × 384 and 384 × 192. This results in 53 K non-zero coefficients which occupies 417 KB of memory. The memorization of neuron activations requires 32-bit per neuron. These are stored in dense vectors and occupy about 182 KB of memory in total (Figure 11). Thus we evaluate that about 10 cores (640 KB of SRAM) are required to train a network on the CIFAR-10 dataset. However, due to the size of the full dataset and the number of operations required to process each image, the training speed of the algorithm might become an issue if the algorithm is parallelized on only 10 cores (the training took approximatively 6 hours on a Tesla p100 GPU with 3,584 cores). For such large datasets, more cores will have to be utilized and a scalable implementation of sparse matrix multiplications will be necessary.

For the TIMIT dataset for speech recognition, a network of 200 recurrent LSTM units was used in Bellec et al. (2018a). LSTM units are more expensive than normal neurons in terms of the number of parameters. More lightweight alternatives have been proposed in Chung et al. (2014), Mikolov et al. (2014), and Bellec et al. (2018b), however, here we consider LSTM networks for benchmarking purposes (Hochreiter and Schmidhuber, 1997). All the parameters of an LSTM are stored in the form of fully connected weight matrices which are constrained to have only 5% of non zero coefficients with DEEP R in Bellec et al. (2018a). The network model is a bi-directional LSTM constituted with two sets of input weights of shape 39 × 800, recurrent weights of shape 800 × 200 and output weights of shape 200 × 39. The 20K non-zero parameters of this sparse LSTM network occupy 158 KB of memory. The network was trained using backpropagation through time (BPTT). At each time step, 6.4 KB were consumed to memorize the activations of the LSTM 1200 gates and 400 LSTM cells and we used 100 unrolled time steps for BPTT so that the activations consumed 640 KB of memory in total (Figure 11). Thus, around 12 cores are needed for training this network. Here, the activations occupy 4 times more memory than the weights due to the storage of unrolled timesteps for BPTT. As a consequence, it might seem that the sparsity in the weights are less relevant in the context of recurrent networks. Interesting possibilities to reduce memory consumption for recurrent neural networks are the quantization and sparsification of the network activity—a specific feature of spiking neural networks. Another alternative is to reduce the number of unrolled timesteps using extensions of BPTT, such as the usage of synthetic gradients (Jaderberg et al., 2016).

This scaling analysis shows that training of models for real-world image and speech recognition will soon be possible on neuromorphic hardware. With the use of DEEP R and a distributed memory storage, a few tens of cores similar to those of our SpiNNaker 2 prototype are enough to store and process the necessary information. With this software solution, such energy-efficient chips will not suffer from their memory restrictions to solve deep learning problems.

### 4.2. Related neuromorphic hardware

A number of papers show implementations of *deep neural networks on neuromorphic hardware*. Most of these use a spiking, pre-trained approach, i.e., the networks are trained in either ANN or SNN fashion, then in the case of ANNs, converted to SNNs, and implemented on the spiking neuromorphic hardware. Examples include TrueNorth (Esser et al., 2015, 2016), SpiNNaker 1 (Jin et al., 2010), the BrainScaleS system (Petrovici et al., 2017; Schmitt et al., 2017), or the Zurich subthreshold systems (Indiveri et al., 2015). Of these, only the last one incorporates some learning, i.e., the last layer of the deep SNN is subject to online supervised learning, with the other layers having pretrained fixed weights. The overall power consumption is not given in Indiveri et al. (2015), but from the power figures of the individual chips, it seems to be below 5 mW. However, as not all weights in the network are trained and no time course for the learning is given, a direct comparison to the energy analysis in section 3.3 is not possible. Stromatias et al. (2015) detailed a non-learning spiking MNIST implementation on SpiNNaker 1. They employed 650 k synapses in a fully connected 4-layer network, which forced the usage of 12 cores and necessitated storing synaptic weights on external DRAM, in turn pushing power consumption to 0.3 W. This clearly shows the advantage of our sparse connectivity approach, which reduces computation and memory requirements concurrently. Esser et al. (2015) classified MNIST on TrueNorth with an accuracy between 92.7 and 99.42% at an energy per image between 0.268 and 108 μ*J*. In contrast, we show 96.6% inference accuracy for MNIST at ≈23 μJ per image classification, which is comparable to TrueNorth. Regarding inference time per image, we achieve 0.1 ms (see section 3.2) and outperform both Stromatias et al. (2015)(tens of milliseconds) and Esser et al. (2015)(1 ms).

#### 4.2.1. Fully programmable, flexible learning function

Almost all of the above examples use pre-trained, fixed-weight deep networks. This is likely due to the fact that neuromorphic systems usually have very narrowly configurable plasticity functions unsuitable for ANNs (Indiveri et al., 2015; Noack et al., 2015). Lately, the notion of a dedicated plasticity processor has gained traction for both mixed signal (Friedmann et al., 2017) and digital systems (Davies et al., 2018). These should in principle offer more freely configurable plasticity. However, both cited examples have restrictions on the state variables accessible to the processor, likely limiting their exploration capability in an ANN context. In the case of the next-generation BrainScaleS system (Friedmann et al., 2017), the processor has access to STDP-type time difference measurements of pre- and post-synaptic spike time differences, firing rates and external signals. The Loihi chip (Davies et al., 2018) is geared toward various kinds of spike time and rate variables as the basis for plasticity. In contrast, we show that the SpiNNaker 2 prototype has very little restrictions in terms of learning rules. Essentially, we took the theoretical learning rule and directly implemented it in hardware with virtually no hardware-caused adaptations. SpiNNaker 2 can incorporate reward factors and access membrane activation as part of the learning as shown here, can do both rate- and spike-based learning, etc.

In the form of *structural plasticity*, the brain is very good at using limited resources to maximum advantage. Neuromorphic systems are usually not a good fit for this learning paradigm, as they contain synaptic matrices fixedly assigned to postsynaptic neurons with only the presynaptic sources to some degree flexible (Noack et al., 2010). Rewiring is possible in such a setup, but has to operate postsynaptic-centric and has a fixed number of synapses per postsynaptic neuron (George et al., 2015). Memristive crossbar arrays are even worse (Du et al., 2015; Mostafa et al., 2015), as both pre- and post-synaptic contacts are fixed and all possible synapses of the rewiring scheme need to be physically instantiated, i.e., using less synapses does not reduce the used resources either in silicon area or in power. In contrast, the flexible memory model we use in this paper on the SpiNNaker 2 prototype allows us to take full advantage of resource reduction.

### 4.3. Efficient DNN processing

Besides applying deep learning on neuromorphic hardware, in recent years, there have been many efforts in building dedicated hardware for efficient DNN processing and developing models with a low memory and compute footprint with negligible loss of performance. The common objective is the application of DNNs for inference in mobile systems with limited memory and computing power (smartphones, robotics, IoT devices, etc.).

Memory reduction is desirable for two reasons: First, on embedded systems the available memory for storing DNN parameters and activations is very limited, and second, the energy cost per memory access can dominate the overall power consumption, e.g., an external memory access can consume more than two orders of magnitude more energy than one multiply-accumulate operation (Horowitz, 2014; Han et al., 2016). A rather old approach for compression of trained networks is *pruning*, which erases less-effective connections from weight matrices. Typically, the pruning is followed by a fine-tuning of the remaining weights. This way, the overall memory per network can be reduced by one order of magnitude or more at negligible loss of network performance (Han et al., 2015). Node pruning (He et al., 2014), weight matrix decomposition (Xue et al., 2013), and filter separation (Bhattacharya and Lane, 2016) are complementary ways to create sparser DNNs with reduced computing requirements. In contrast, *quantization* uses lower bit width numbers to store the multifarious parameters of a DNN: Previous works (Courbariaux et al., 2016; Esser et al., 2016; Hubara et al., 2018) have quantized the weight matrices and layer activations to reduce the memory footprint. This reduces the memory consumption of a network for testing but not during training as it is necessary to propagate full-precision gradients during training of those neural networks.

At the same time, there is active research in the *design of efficient models* targeted for mobile applications, e.g., MobileNets (Howard et al., 2017) are light-weight models using depth-wise separable convolutions allowing to trade off computational complexity against model accuracy through hyperparameters. Similarly, ShuffleNet (Zhang et al., 2017) and CondenseNet (Huang et al., 2017) apply sparsely connected convolutions between feature maps in CNNs to reduce the compute load. All these models have in common that they are optimized for inference in mobile devices but need to be trained in data-centers.

#### 4.3.1. Hardware for efficient inference of DNNs

Typically employs many multiply-accumulate (MAC) units as this is the main operation in DNNs. Often, low-bit integer operations are implemented as they require less power and silicon area than floating point units Horowitz (2014). To achieve high throughput and energy-efficiency, hardware architectures have an optimized data flow that reuses data (weights, feature maps) as much as possible and avoids accessing off-chip memory (Sze et al., 2017). While most DNN hardware systems directly benefit from quantization and efficient model architectures, sparse networks require dedicated accelerators (Han et al., 2016). Instead, sparse activity (neurons with output 0) can be exploited more easily (Aimar et al., 2017; Moons et al., 2017). State-of-the-art DNN processors achieve an energy-efficiency in the order of 10 TOPS/W with few-bit integers (Lee et al., 2018) or even more than 500 TOPS/W for binarized neural networks in mixed-signal hardware (Bankman et al., 2018). Although these systems are orders of magnitude more efficient than our prototype system (see section 3.3), their application is limited to inference and on-chip training is not possible.

### 4.4. Deep learning with a small memory footprint

The training of machine learning models with memory constraints is especially useful for low-power mobile and edge devices: For example, in the context of Internet of Things, smart sensors can employ deep learning to extract relevant features from raw data. While pre-trained models can be loaded to the sensors, an in-place training and fine-tuning allow for adapting the model to the environment or changes of sensor characteristics (e.g., from aging). Aside from that, simple robots interacting with the environment can learn autonomously to perform specific tasks. Especially reinforcement learning is highly applicable to the device as training examples are immediately available. Only a few algorithms for deep learning with a small memory footprint have been proposed. (Cheng et al., 2015; Sindhwani et al., 2015) replaced matrix multiplications with structured operations that require less memory. A method called WAGE has been developed to train DNNs with low bitwidth integers at all stages, including gradients and backpropagated errors (Wu et al., 2018). Very recently, training methods were also ported onto specific hardware systems: Gonugondla et al. (2018) present a deep in-memory architecture with on-chip training, which is primarily useful to compensate for PVT variations of the analog circuits.

In this article, we presented a scalable implementation of a 4-layer DNN for digit recognition on a SpiNNaker 2 prototype chip using the DEEP R algorithm (Bellec et al., 2018a). This show that rewiring is a viable strategy for neuromorphic hardware with strong memory constraints. The training was achieved with less than 52 KB overall memory while being two orders of magnitudes more energy-efficient than a CPU. In the future, a combination of the proposed approach with low bit-width training methods such as WAGE (Wu et al., 2018) could be used to further reduce the memory requirements during training and inference.

## Data availability statement

The X86 implementation of the DEEP R algorithm used in this paper will be made available at publication of the paper.

## Author contributions

CL performed the experiment on the prototype chip and measured the metrics with the aid of BV. GB and RL conceived and designed the DEEP R algorithm with the help of DK. JP and FN developed the hardware accelerator of exponential function and random number generation. SH and SF contributed to the chip design. RL, WM, and CM supervised the findings of this work. All authors discussed the results and contributed to the final manuscript.

### Conflict of interest statement

The authors declare that the research was conducted in the absence of any commercial or financial relationships that could be construed as a potential conflict of interest.
